# LINE-1 global DNA methylation, iron homeostasis genes, sex and age in sudden sensorineural hearing loss (SSNHL)

**DOI:** 10.1186/s40246-023-00562-9

**Published:** 2023-12-14

**Authors:** Veronica Tisato, Alessandro Castiglione, Andrea Ciorba, Claudia Aimoni, Juliana Araujo Silva, Ines Gallo, Elisabetta D’Aversa, Francesca Salvatori, Chiara Bianchini, Stefano Pelucchi, Paola Secchiero, Giorgio Zauli, Ajay Vikram Singh, Donato Gemmati

**Affiliations:** 1 Department of Translational Medicine, University of Ferrara, 44121 Ferrara, Italy; 2 LTTA Centre, University of Ferrara, 44121 Ferrara, Italy; 3 University Strategic Centre for Studies on Gender Medicine, University of Ferrara, 44121 Ferrara, Italy; 4 Audiology Department, Örebro University Hospital, 70210 Örebro, Sweden; 5 Department of Neurosciences, University Hospital of Ferrara, 44121 Ferrara, Italy; 6 Department of Environmental and Prevention Sciences, University of Ferrara, 44121 Ferrara, Italy; 7 Department of Chemical and Product Safety, German Federal Institute for Risk Assessment (BfR), 10589 Berlin, Germany; 8 Centre Haemostasis and Thrombosis, University of Ferrara, 44121 Ferrara, Italy

**Keywords:** Epigenomics, Epigenetics, Epidrugs, Iron, LINE-1 methylation, Oxidative stress, Pharmacogenomics, Pharmacogenetics, SSNHL

## Abstract

**Background:**

Sudden sensorineural hearing loss (SSNHL) is an abrupt loss of hearing, still idiopathic in most of cases. Several mechanisms have been proposed including genetic and epigenetic interrelationships also considering iron homeostasis genes, ferroptosis and cellular stressors such as iron excess and dysfunctional mitochondrial superoxide dismutase activity.

**Results:**

We investigated 206 SSNHL patients and 420 healthy controls for the following genetic variants in the iron pathway: *SLC40A1* − 8CG (ferroportin; FPN1), *HAMP* − 582AG (hepcidin; HEPC), *HFE* C282Y and H63D (homeostatic iron regulator), *TF* P570S (transferrin) and *SOD2* A16V in the mitochondrial superoxide dismutase-2 gene. Among patients, *SLC40A1* − 8GG homozygotes were overrepresented (8.25% vs 2.62%; *P* = 0.0015) as well *SOD2* 16VV genotype (32.0% vs 24.3%; *P* = 0.037) accounting for increased SSNHL risk (OR = 3.34; 1.54–7.29 and OR = 1.47; 1.02–2.12, respectively). Moreover, LINE-1 methylation was inversely related (*r*^*2*^ = 0.042; *P* = 0.001) with hearing loss score assessed as pure tone average (PTA, dB HL), and the trend was maintained after *SLC40A1* − 8CG and *HAMP* − 582AG genotype stratification (Δ_*SLC40A1*_ = + 8.99 dB HL and Δ_*HAMP*_ = − 6.07 dB HL). In multivariate investigations, principal component analysis (PCA) yielded PC1 (PTA, age, LINE-1, *HAMP*, *SLC40A1*) and PC2 (sex, *HFE*_*C282Y*_, *SOD2, HAMP*) among the five generated PCs, and logistic regression analysis ascribed to PC1 an inverse association with moderate/severe/profound HL (OR = 0.60; 0.42–0.86; *P* = 0.0006) and with severe/profound HL (OR = 0.52; 0.35–0.76; *P* = 0.001).

**Conclusion:**

Recognizing genetic and epigenetic biomarkers and their mutual interactions in SSNHL is of great value and can help pharmacy science to design by pharmacogenomic data classical or advanced molecules, such as epidrugs, to target new pathways for a better prognosis and treatment of SSNHL.

## Introduction

Sudden sensorineural hearing loss (SSNHL) etiopathogenesis is not completely known and various genetic and environmental causes cooperate in its establishment. It is considered a critical clinical and public health issue [1–6], characterized by a mean annual incidence of 27 per 100,000 people in the USA reaching 77 per 100,000 for those older than 65 years [7]. By a gender point of view, there is slight greater male preponderance (1.07:1; M/F ratio), higher in cases over 65 or older (1.30:1; M/F ratio). According to the WHO, by 2050 about 2.5 billion people might experience some degree of hearing loss (HL) and the National Academies of Sciences Engineering and Medicine expects HL becomes the fifth most common disability [8].

Basically, among the proposed factors involved in HL, a genetic etiology is the most commonly considered [9–15] accounting for more than 50% of all cases [16]. Globally, more than 150 genetic loci have been significantly linked to SNHL [17] which defects often cause the degeneration of the inner ear sensory epithelia or the hair cells in the Corti organ affecting normal hearing ability [18–20]. Moreover, active researches have also been directed toward selected gene polymorphisms affecting crucial pathways mainly accounting for thrombosis and inflammation (see rev. of literature in [21]) also considering viral infections as recently reported after COVID-19 or anti-SARS-CoV-2 vaccination hypothesizing an increased number of SSNHL reported after the outbreak [22, 23]. Oxidative-stress-associated genes prevent damages to cellular components by counteracting superoxide radicals (i.e., ROS), and local excess of iron by contributing to ROS production and enhancing redox cycling is particularly detrimental for any kind of cell [24–26] and epithelium in acute or chronic diseases from the nervous system [27, 28] to the skin apparatus [29–31]. Accordingly, local iron dyshomeostasis and ROS unbalancing due to gene polymorphisms, by affecting sensitive cells as those of the ear sensory epithelia, have been proposed as mechanism predisposing to SSNHL [32–35]. Moreover, ferroptosis, a novel form of non-apoptotic regulated cell death connected to intracellular iron overloading and iron-dependent lipid peroxidation, has been proposed as a mechanism associated with hearing loss [36].

Epigenetics and genetics closely cooperate in revealing the basic mechanisms of complex diseases to find novel therapeutic targets and informative prognostic indicators [37–41]. Epigenetic status may change according to the environmental conditions experienced by individual as aging, lifestyle, infections, toxic exposure and concomitant pathologies, for this reason epigenetic markers can be considered either as indicators of a disease or be themself causative of the pathologic condition [42, 43]. Moreover, inherited predispositions or gene mutations may paint the individual epigenetic landscape and be responsible for the onset of several pathologies as cancer, neurological diseases, pregnancy loss and delayed wound healing [44–46]. DNA methylation, iron homeostasis and local iron levels are closely related [47, 48], and all of them may finely tune the expression of iron-driven genes as *SLC40A1* and *HAMP*, as recently proposed in studies aimed at discovering relationships between iron homeostasis pathway and DNA methylation trajectories also considering the potential role of ferroptosis in complex diseases [49, 50]. Interestingly, the status of LINE-1 methylation and hearing loss share strong peculiar susceptibilities as environmental exposure to toxic metals, pesticides, noise and pollution [51–53], with direct associations with healthier lifestyle and inverse associations with inflammation, C-reactive protein (CRP), and oxidative stress, basically considering altered global DNA methylation profiles associated with various complex diseases and aging [54, 55].

Accordingly, it is known that metal overload and epigenetic changes may affect the cochlea or the sensorial epithelium being involved in various forms of SNHL [36, 56, 57], as previously reported by our group describing how iron homeostasis genes predispose to idiopathic SSNHL [32, 58]. Finally, to analyze the mutual active interactions existing between iron balance, oxidative stress and methylation, in the present study we investigate how these pathways might synergize or cooperate in tuning the molecular mechanisms of hearing loss in a cohort of SSNHL patients.

## Materials and methods

### Study design and samples collection

A retrospective study aimed at assessing genetic and epigenetic predispositions and mutual interactions to SSNHL has been performed in a cohort of 206 patients belonging to the files of our previous studies on hearing loss (DOR1759543/17) [32, 59–61]. Considering the rationale that iron SNPs significantly balance and regulate iron homeostasis and that hearing loss is exacerbated by iron-driven inflammation and aging, the existence of epigenetics link between these two pathways prompted us to molecularly investigate the whole cohort of SSNHL cases. The research was conducted at the Audiology Department of the University Hospital of Ferrara, in compliance with the Helsinki Declaration, the retrospective/observational study did not affect patient’s care, and cases were informed on the research project during the visit giving the consent in order to participate to the study [32].

### Cases and controls characteristics

Table 1 shows the main clinical and demographic characteristics of SSNHL cases and matched controls. Patients and controls belong to the files of our previous studies on hearing loss [32, 61]. They have also been assessed to investigate common inherited prothrombotic predispositions within the MAGISTER study [62]. Globally, 206 patients (105 females and 101males), affected by idiopathic SSNHL, were enrolled for this study. SSNHL was defined as a sudden hearing loss (≥ 30 dB HL), within 3 consecutive frequencies, developing over 72 h [63]. The distribution of HL score among the cohort of patients was as follow: mild 21%; moderate 47%; severe 17.0%, profound 15.5%. Patients underwent to a clinical interview with a complete audiological assessment, including micro-otoscopy, tonal and speech audiometry, impedancemetry, auditory brainstem responses (ABRs) and MRI with gadolinium to rule out retrocochlear pathology. Exclusion criteria were specific causes of sudden hearing impairment such as meningitis, traumas or surgery outcomes and complications as previously described [32]. The control group consisted of 420 healthy volunteers with no personal or familial history of previous SSNHL, and they were completely matched with the case group by sex, age and ethnicity.

**Table 1:** Main clinical and demographic findings of cases and controls

	Cases *n* = 206	Controls *n* = 420	*P*-value
Age			
Mean ± SD	62.5 ± 14.5	61.5 ± 15.7	n.s
Male/Female	101/105	205/215	
Male %	49.03	48.8	n.s
Hearing Loss			
Mild, *n*, %	43 (20.8)	–	–
Moderate, *n*, %	96 (46.6)	–	–
Severe, *n*, %	35 (17.0)	–	–
Profound, *n*, %	32 (15.5)	–	–

### Genotyping analyses

Detection of the selected gene variants was performed by PCR amplification using the Universal Master Mix (Sentinel Diagnostics, Milan, Italy), and the PCR cycles and protocols were as previously described [32, 64, 65] for the different SNPs investigated: *SLC40A1 *− 8CG (rs11568351), *HAMP *− 582AG (rs10421768), *HFE* C282Y (rs1800562), *HFE* H63D (rs1799945), *TF* P570S (rs1049296) and *SOD2* A16V (rs4880). PCR amplification was performed in a PTC-200 thermal cycler (MJ Research, Inc., Watertown, MA, USA), and the SNP detection was according to Pyromark ID System (Biotage AB Uppsala, Sweden) by using standard procedures selected to have at least 98.0% compatibility score as previously described [32]. Haplotypes were confirmed by re-genotyping approximately 20% of randomly selected samples among each different genotype group for each specific SNP by means of enzymatic restriction of PCR amplicons. There were no discrepancies between genotypes determined in duplicate and/or by different methods. Known genotypes were used as internal controls.

### LINE-1 methylation by pyrosequencing

Extracted DNA (500 ng) from each sample (DNA isolation Qiagen, Hilden, Germany) was bisulfite-converted by EpiTect 96 Bisulfite Kit (Qiagen, Hilden, Germany) in a final volume of 50 µl, according to the manufacturer’s recommendation. Converted DNA was then stored at − 20 °C. The long interspersed nucleotide element 1 (LINE-1) was analyzed as surrogate of genome-wide DNA methylation. A 150-bp nucleotide sequence containing five CpGs sites (+ 306 to + 364; GenBank accession number: X58075) was PCR amplified by Pyromark PCR kit (Qiagen, Hilden, Germany), using specific LINE-1 primers: (Fw: 5′-TTTTGAGTTAGGTGTGGGATATA-3′; Rev: 5′Bio-AAAATCAAAAAATTCCCTTTC-3′) and SureCycler_8800 (Agilent Technologies, Mulgrave, AU). Thermocycling protocol was as follows: one initial step 95 °C, 15 min; followed by 38 cycles of 94 °C, 30 s; 55 °C, 30 s; 72 °C, 30 s; plus, final 10-min extension at 72 °C. PCR specificity was verified by 8.5% PAGE. Methylation of CpG dinucleotides was finally analyzed by PyroMark Q96 ID (Qiagen, Hilden, Germany), using a specific sequencing primer (5′-AGTTAGGTGTGGGATATAGT-3′), and calculated as the percentage of cytosine nucleotides relative to the sum of cytosine and thymine nucleotides in a given position by Pyromark Q96 software v1.01. Overall LINE-1 DNA methylation was calculated as the mean of the C percentage of the CpGs sites analyzed.

### Statistical analyses

Statistical analyses were performed using SPSS Statistics version 22 (SPSS Inc., Chicago, IL, USA) and MedCalc version 20.112 (MedCalc Software Ltd.). All figures were produced by GraphPad Prism9 (GraphPad Software, Inc., San Diego, California USA), unless otherwise specified. The Kolmogorov–Smirnov test was used to verify variables normal distribution. Normally distributed data are presented as mean and SD and Student’s t-test to compare differences in normal variables between two independent groups. The Mann–Whitney U test was applied for the allele and genotype comparisons between cases and controls, and the dominant and recessive models have been applied. Genotypes, methylation, PTA, sex and age were subjected to Principal Component Analysis (PCA). SNPs were scored 1, 2 and 3 to represent common homozygous, heterozygous and rare homozygous variant, respectively, to indicate an increasing number of the variant allele (i.e., 0, 1 and 2, respectively). Age, methylation and PTA were centered and scaled before PCA according to the formula (*x*-value − mean value)/SD [*Z* =  (*x* − *μ*)/*σ*]. Collinearity diagnostics evaluation was assessed by variance inflation factor (VIF), and values below 5.0 have been considered as threshold. PCA was performed by retaining those PCs with eigenvalues exceeding 1.0. Eigenvector of independent variables with absolute value exceeding 0.3 (+ or −) was included. Variables with a loading above the cut-off point 0.3 were considered to be dominant in a component. Scores for each PC for each individual were extracted by using regression models. Retained PCs were computed in logistic regression analysis for the different PTA (dB HL) scores (mild = 0 *versus* moderate/severe/profound = 1; and mild/moderate = 0 *versus* severe/profound = 1) *versus* the selected PC. P-values were two-sided with threshold for statistical significance fixed to *P* ≤ 0.05.

## Results

### Genotype single analysis, LINE-1 methylation and correlation assessment

Table 2 shows the significant genotype distributions of the SNPs investigated and the crude ORs calculation in the SSNHL cases and healthy controls. The significant overrepresentation of the homozygous *SLC40A1* -8GG genotype in cases compared to controls (8.25% vs 2.62%, respectively; *P* = 0.0015) accounted for an increased SSNHL risk in -8GG carriers (OR = 3.34; 1.54–7.29) assessed as recessive model. Similarly, *SOD2* 47C > T responsible for the amino acid change A16V yielded an overrepresentation of the homozygous 16VV (32.0% vs 24.3%, respectively; *P* = 0.037) accounting for an increased SSNHL risk in 16VV carriers (OR = 1.47; 1.02–2.12) assessed as recessive model. The remaining SNPs did not reach significant differences in the three genetic comparison models applied.

**Table 2:** Genotype distribution and crude ORs

	*SLC40A1* − 8CG (rs11568351)			*SOD2 *A16V c.47C > T (rs4880)		
Cases *n* = 206 (%)	CC 127 (61.6)	CG 62 (30.1)	GG 17 (8.25)	CC 43 (20.9)	CT 97 (47.1)	TT 66 (32.0)
Genotype distribution (P)	**0.005**			0.09		
OR D-model (P)	1.18 (0.83–1.77); n.s			1.31 (0.88–1.96); n.s		
OR R-model (P)	**3.34 (1.54–7.29); 0.0015**			**1.47 (1.02–2.12); 0.037**		
OR Allele (P)	**1.31 (1.01–1.79); 0.037**			**1.28 (1.02–1.63); 0.036**		
Controls *n* = 420 (%)	CC 276 (65.7)	CG 133 (31.7)	GG 11 (2.6)	CC 108 (25.7)	CT 210 (50.0)	TT 102 (24.3)

OR D-model and R-model indicate dominant and recessive model comparison, respectively. The significant* P*-values and ORs are marked in bold

Table 3 shows the global DNA methylation, assessed as mean percentage of LINE-1 methylation, and the average degree of HL, assessed as PTA (dB HL), stratified by the genotype distribution of the six SNPs investigated. Methylation score stratified by genotypes yielded subtle not significant differences in *SLC40A1* gene (CC vs GG, Δ = − 1.9%) and in *HFE* H63D (HH vs HD + DD, Δ =  + 2.3%).

**Table 3:** LINE-1 methylation and PTA score in SSNHL cases stratified by genotypes

	*SLC40A1* − 8CG (rs11568351)			*HAMP* − 582AG (rs10421768)			*HFE C282Y* c.845G > A (rs1800562)		*HFE H63D* c.187C > G (rs1799945)			*TF* P570S c.1765C > T (rs1049296)			*SOD2 A16V* c.47C > T (rs4880)		
Cases *n* = 206 (%)	CC 127 (61.6)	CG 62 (30.1)	GG 17 (8.2)	AA 109 (52.9)	AG 82 (39.8)	GG 15 (7.3)	GG 199 (96.6)	GA 7 (3.4)	CC 149 (72.3)	CG 55 (26.7)	GG 2 (0.97)	CC 145 (70.3)	CT 53 (25.7)	TT 8 (3.9)	CC 43 (20.9)	CT 97 (47.1)	TT 66 (32.0)
LINE-1*%* mean ± SD	87.9 ± 3.9	87.09 ± 3.6	86.0 ± 4.4	87.76 ± 3.5	87.63 ± 4.1	87.74 ± 3.9	87.5 ± 3.8	86.1 ± 3.6	86.7 ± 3.8	88.9 ± 3.0	91.5 ± 1.7	87.3 ± 3.9	87.7 ± 3.7	87.4 ± 3.6	86.6 ± 3.8	87.7 ± 4.0	87.6 ± 3.5
PTA *dB* mean ± SD	59.36 ± 22.5	62.3 ± 23	68.35 ± 22.1	62.1 ± 22.4	60.45 ± 21.2	56.03 ± 31.5	61.11 ± 22.9	57.53 ± 20.3	60.99 ± 22.1	61.22 ± 24.4	54.35 ± 10.5	61.66 ± 22.5	59.38 ± 23.3	59.46 ± 23.1	61.09 ± 15.1	61.19 ± 25.6	61.36 ± 22.3

Similarly, dB HL stratified by *SLC40A1,* or *HAMP* gene variants yielded interesting different findings, showing opposite trends in the dB HL score (i.e., *SLC40A1*: CC < CG < GG; CC vs GG, Δ =  + 8.99 dB) and (i.e., *HAMP*: AA > AG > GG; AA vs GG, Δ = − 6.07 dB).

**Fig. 1:**
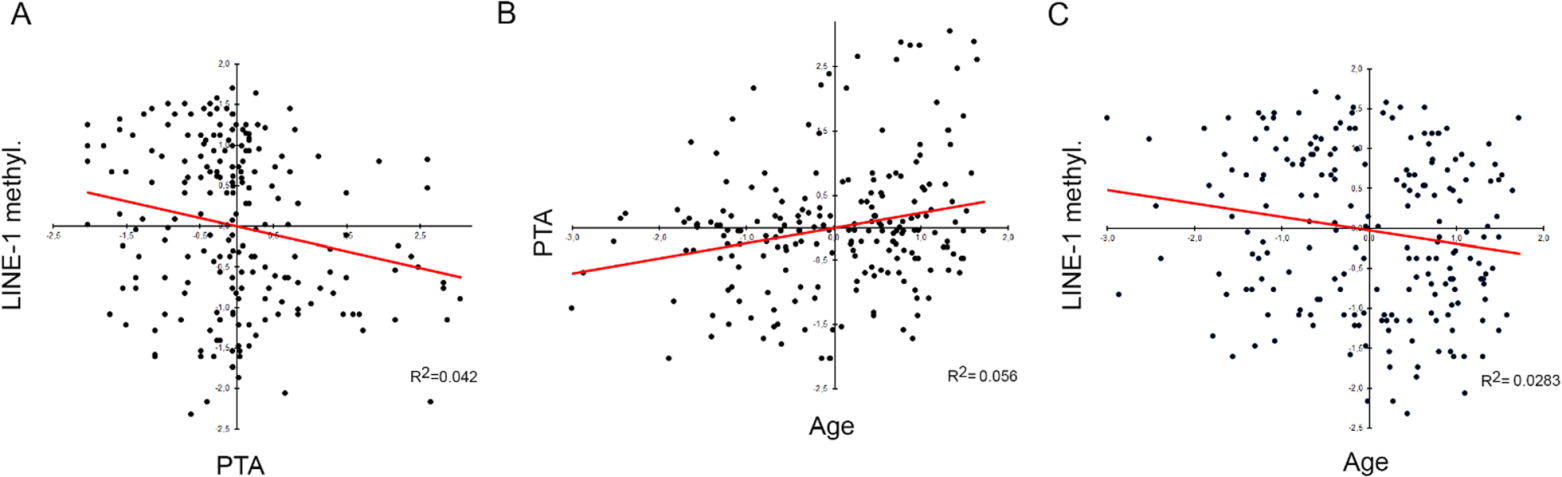
Correlation analysis in the whole cohort of patients. Scatter plots of the correlation between PTA and LINE-1 methylation (**A**), age and PTA (**B**), age and LINE-1 methylation (**C**). Variables were centered and scaled as described in Materials and Methods section. Each panel shows the specific regression line in red and the *R*^2^ coefficient

In an explorative approach, in order to deepen any possible association among genotypes, LINE-1 methylation, and PTA scores, we firstly correlated methylation with PTA and afterward stratified regression analyses by the genotypes of *SLC40A1* and *HAMP* genes, being those with the widest PTA gaps among appreciable number of the three classes of genotype. A significant inverse relation between methylation and PTA resulting in an increased hearing loss severity as the global DNA methylation decreased was detected (*r*^*2*^ = 0.042; *P* = 0.001) (Fig. 1A). Considering that hearing ability normally decreases as age increases and that aging was associated with methylation lowering, due to loss of function in DNMT enzymes, we further determined correlation analyses comparing PTA *versus* age and methylation *versus* age. Accordingly, we found a significant loss of hearing ability (assessed as PTA) and a significant decreasing of mean methylation as the age of patients increased (*r*^*2*^ = 0.056; *P* = 0.001 and *r*^*2*^ = 0.0283; *P* = 0.001, respectively) (Fig. 1B, C).

**Fig. 2:**
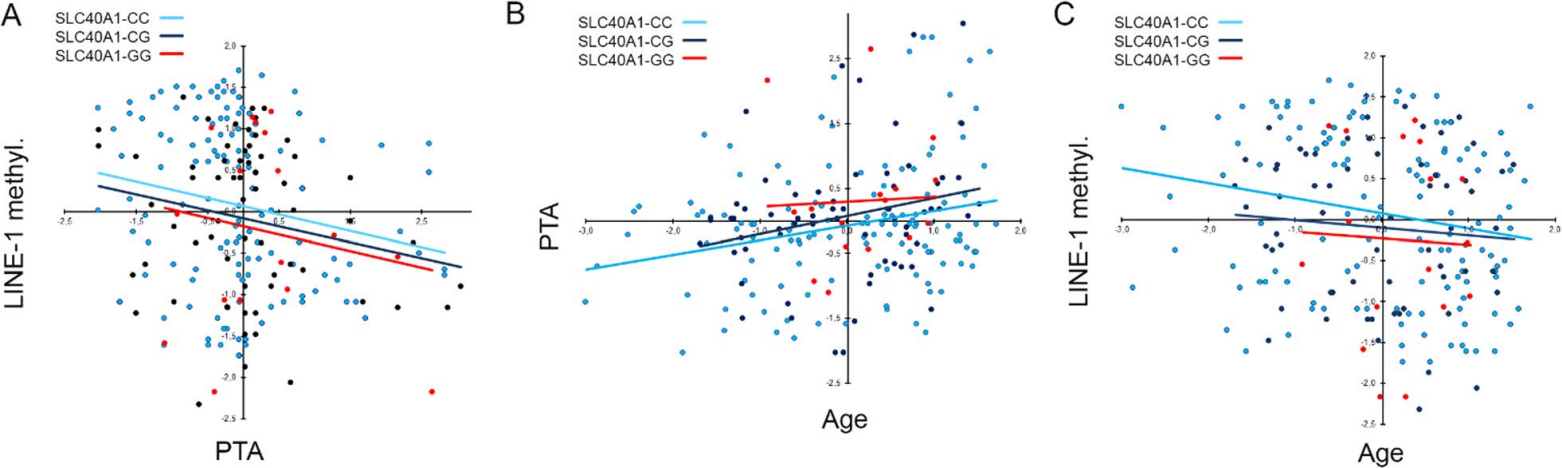
Correlation analysis in the whole cohort of patients stratified by *SLC40A1* gene variant. Scatter plots of the correlation between PTA and LINE-1 methylation (**A**), age and PTA (**B**), age and LINE-1 methylation (**C**). Variables were centered and scaled as described in Materials and Methods section. Each panel shows the specific regression lines, according to the indicated *SLC40A1* genotype

Interestingly, the same regression analyses, assessed after genotype stratification, ascribed to the *SLC40A1* − 8G allele greater direct correlations in determining the inverse correlation between LINE-1 methylation and PTA (Fig. 2A), the age-related lowering of hearing ability (Fig. 2B) and the age-related lowering of LINE-1 methylation (Fig. 2C) as the number of the -8G alleles increased in the genotype of patients. The same approach applied to the *HAMP* − 582AG variant yielded completely opposite trends though less robust (data not shown). Combined genotype sub-analyses could not be performed due to paucity of the rare genotypes, hypothesizing that the greatest effect could be observed comparing those cases carrying the pure homozygous genotypes. The remaining stratifications did not yield comparable trends as those for *SLC40A1* gene.

### PCA and logistic regression analysis of the principal components (PCs)

**Fig. 3.**
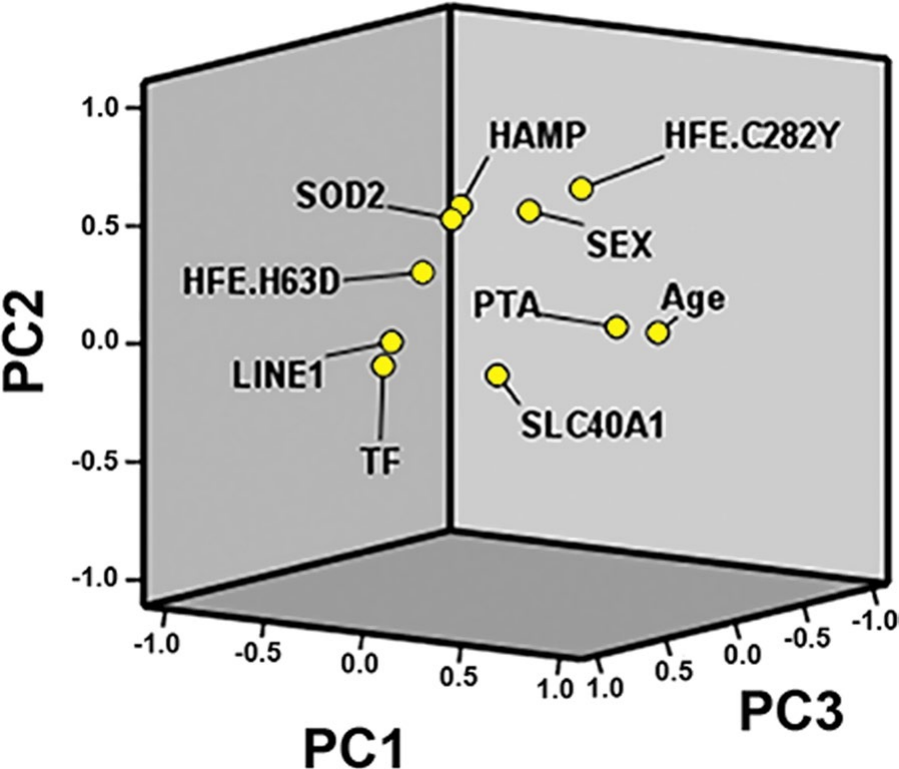
3D plot of principal component analysis for the computed 10 variables: PC1, PC2 and PC3 loadings. Plotted by SPSS (Statistics version 22)

To explore and disclose possible relationships between the complex clinical phenotype of SSNHL and genetic/epigenetic findings, we performed a series of PCA and logistic regression analyses. Firstly, we analyzed by PCA the six SNPs, the LINE-1 mean methylation and the PTA scores also accounting for sex and age in the cohort of patients. The Bartlett’s test was *P* = 0.01, confirming that PCA test was appropriate. Accordingly, the first five PCs selected explained more than 60% of the total variance, and considering only those variables with eigenvector value exceeding 0.3 (+ or −), they mainly accounted for: PC1 (PTA, age, LINE-1, *HAMP*, *SLC40A1*), PC2 (sex, *HFE*_*C282Y*_, *SOD2, HAMP*), PC3 (*TF, SLC40A1, HFE*_*C282Y*_), PC4 (*HFE*_*H63D*_*, SOD2, HFE*_*C282Y*_*, TF*) and PC5 (*SLC40A1, SOD2*, age, *HFE*_*C282Y*_*,* LINE-1) as summarized in Table 4. Finally, the 3D loading plot displays how the ten computed variables allocate along with the first three selected PCs overall explaining more than 40% of dataset intergroup variability (Fig. 3).

**Table 4:** Principal components composition PTA included

Variables	PCs				
	PC1	PC2	PC3	PC4	PC5
Sex	0.240	**0.575**	0.247	0.110	0.134
Age	**0.622**	0.041	− 0.141	0.152	**0.378**
*SLC40A1*	**0.302**	− 0.072	**0.557**	− 0.130	**− 0.519**
*TF*	− 0.199	− 0.065	**0.677**	**− 0.302**	0.058
*HFE*_*H63D*_	− 0.266	0.270	0.296	**0.718**	− 0.145
*HFE*_*C282Y*_	0.059	**0.559**	**− 0.390**	**− 0.361**	**− 0.371**
*HAMP*	**− 0.364**	**0.482**	− 0.120	0.200	− 0.189
*SOD2*	− 0.145	**0.503**	0.255	**− 0.404**	**0.458**
PTA	**0.630**	0.111	0.170	0.248	0.129
LINE-1	**− 0.615**	− 0.101	0.022	0.118	**0.328**

In bold the main loadings exceeding the cut-off > 0.30 or < − 0.30

**Fig. 4.**
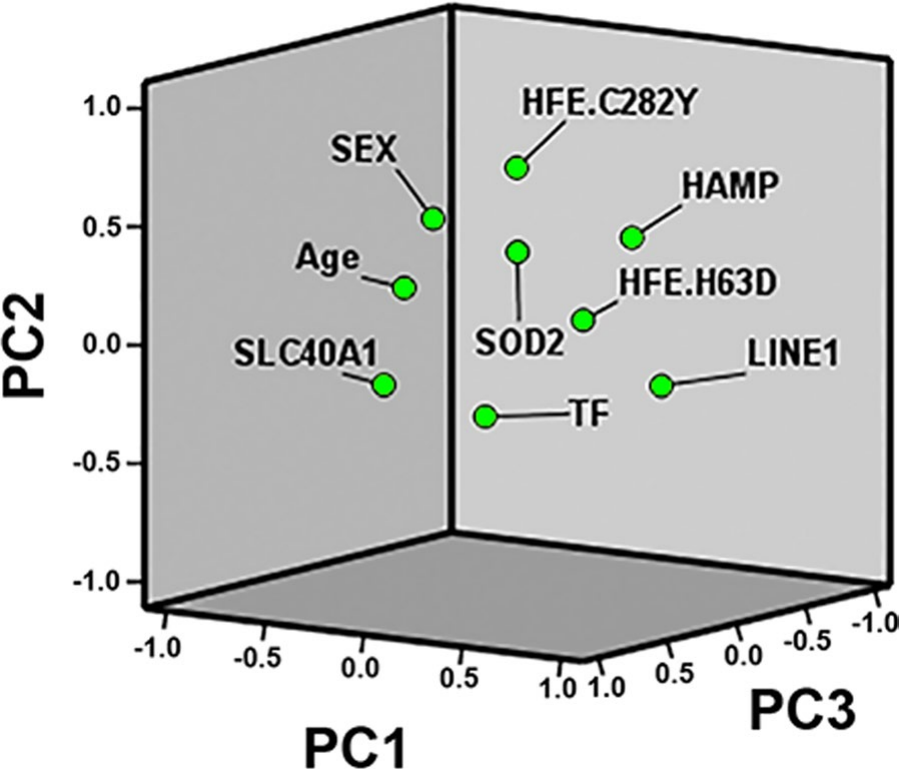
3D plot of principal component analysis for the computed 9 variables: PC1, PC2 and PC3 loadings. Plotted by SPSS (Statistics version 22)

By excluding PTA among the group of variables, PCA now yielded the following five PCs overall explaining about 65% of dataset intergroup variability: PC1 (LINE-1, age, *HAMP, HFE*_*H63D*_, *SLC40A1)*, PC2 (*HFE*_*C282Y*_*,* sex, *HAMP, SOD2*), PC3 (*TF, SLC40A1,* sex, *SOD2*), PC4 (*HFE*_*H63D*_*, SOD2,* sex, *TF*) and PC5 (*SLC40A1,* age, *SOD2*, *HFE*_*C282Y*_*, HAMP,* LINE-1) as summarized in Table 5. Finally, the 3D loading plot displays how the nine computed variables allocate along with the first three selected PCs overall explaining more than 55% of dataset intergroup variability (Fig. 4).

**Table 5:** Principal components composition excluding PTA

Variables	PCs				
	PC1	PC2	PC3	PC4	PC5
Sex	− 0.136	**0.532**	**0.415**	**0.320**	0.292
Age	**− 0.600**	0.127	− 0.040	0.193	**0.405**
*SLC40A1*	**− 0.343**	− 0.152	**0.619**	0.084	**− 0.454**
*TF*	0.280	− 0.230	**0.631**	**− 0.377**	− 0.017
*HFE*_*H63D*_	**0.444**	0.123	0.156	**0.735**	− 0.045
*HFE*_*C282Y*_	− 0.113	**0.667**	− 0.158	− 0.279	**− 0.363**
*HAMP*	**0.472**	**0.431**	− 0.156	0.067	**− 0.317**
*SOD2*	0.250	**0.421**	**0.355**	**− 0.441**	**0.392**
LINE-1	**0.621**	− 0.180	− 0.155	− 0.035	**0.334**

In bold the main loadings exceeding the cut-off > 0.30 or < − 0.3

In the attempt to associate PCs with the risk of developing more severe SSNHL, we considered PTA scores as dependent variables and PCs as independent variables in a logistic regression model. We found significant inverse association of PC1 with the risk of mild HL when compared with the remaining HL degrees (i.e., moderate, severe, profound) and with mild/moderate HL *versus* severe/profound degrees (OR: 0.60; 0.42–0.86; *P* = 0.0006 and OR: 0.52; 0.35–0.76; *P* = 0.001, respectively).

## Discussion

SSNHL etiology remains in part unknown; however, recent findings from genetic and epigenetic investigations suggest possible underlying mechanisms in the auditory system [66–68]. Aging, inflammation and vasculature anomalies may change local vessel permeability and stasis causing endothelium and RBC damage, micro-thrombosis, local iron overload and increased oxidative stress [69, 70]. These conditions may affect the cochlea, the sensorial epithelium or the blood–labyrinth barrier causing in turn SNHL [21, 71, 72]. Basically, balanced DNA methylation and iron homeostasis cooperate in tandem to maintain appropriate organ functions and avoid ferroptosis and iron-induced epigenetic abnormalities as recently confirmed in brain, bone marrow, mitochondria and auditory system [36, 49, 66, 73]. Moreover, a strong relation of global and gene-specific DNA methylation with iron homeostasis is further supported by the observation that the rate of methylated cytosines was higher in individuals with iron overload, and whole-genome bisulfite sequencing highlighted epigenetic changes in several candidate genes including *HFE, SLC401, TFRC*, suggesting that DNA methylation directly affected iron content by tuning specific iron-sensitive genes [47]. Finally, brain and CNS are highly susceptible to iron-related cell death and interventions targeted to mitigate ferroptosis demonstrated improved recovery in animal models of cerebral aneurism hemorrhage and reduced iron-mediated cytotoxicity in cochlear hair cells, considering DNA methylation as an informative biomarker [49, 74].

In the present paper, we explored the mutual interplay among global DNA methylation (LINE-1) and the main gene variants involved in iron homeostasis and redox status in relation to the degree of HL in a cohort of SSNHL patients by single and combined PCAs. The main findings of this investigation are the significant direct correlation between age and the degree of HL, and the significant inverse correlations between age and LINE-1 methylation furtherly summarized in a significant inverse correlation between LINE-1 methylation and the degree of HL, ascribing to methylation a putative role of causative factor or of informative biomarker for SSNHL.

On the other hand, the less apparent connection found between genotypes and methylation was instead strengthened by considering the strong inverse correlation observed between LINE-1 methylation and PTA furtherly confirmed in a stepwise fashion when stratified by *SLC40A1* genotypes. Globally, the genotype findings are mainly focused on the *SLC40A1*-*HAMP* axis showing on the one hand the significant increased SSNHL risk (*P* = 0.0015) in patients carrying the *SLC40A1* − 8GG homozygous genotype and on the other hand an interesting opposite trend of PTA scores stratified by *SLC40A1* or *HAMP* genes as the copy of the polymorphic allele increased in the genotype of patients (i.e., Δ_*SLC40A1*_: + 8.99 dB; Δ_*HAMP*_: − 6.07 dB, respectively). The opposite observed trends realistically reflect the molecular mechanisms of ferroportin and hepcidin in the balancing of iron homeostasis and overload, the former by opening the cellular iron-gate and the latter by limiting ferroportin-driven iron release according to the systemic and local iron availability [75].

Moreover, *SOD2* 47C > T was the next SNP among those analyzed associated with increased SSNHL risk in a recessive model (*P* = 0.037) ascribing to the *SOD2* 16VV homozygotes a moderate increased risk. Less robust was instead the different mean of LINE-1 methylation comparison stratified by genotypes;*SLC40A1 *− 8CG opposite genotypes accounted for Δ_*SLC40A1*_ = − 1.9%, and *HFE * H63D for Δ_*H63D*_ =  + 2.34% by the dominant model.

Although the subtle gaps in the magnitude of dB HL stratified by genotypes, a hypothesized mechanism could be considered reliable in view of the antagonistic role of *SLC40A1* and *HAMP* genes on cell iron accumulation in response to the available iron burden in health and disease [75–78], also considering that iron excess generates high ROS burden considered detrimental if not properly neutralized by mitochondrial superoxide dismutase as in the presence of the loss of function *SOD2* V16 allele [79, 80].

According to the iron hypothesis, a suboptimal hepcidin–ferroportin axis takes strong part in neurodegeneration [24, 25, 27, 81, 82], and together with other iron homeostasis and oxidative stress genes, ROS unbalancing may have a detrimental role also on the auditor system [33, 34, 58]. Ferroportin is the unique cellular iron exporter and is post-transcriptionally regulated by hepcidin, and then, a decreased ferroportin expression reduces external iron export and maintains accumulation of iron in the cell, while hepcidin expression is controlled by the effects of iron overload and inhibits ferroportin by cell internalization [83]. Iron excess in turn exacerbates local oxidative stress, and if not properly controlled, as in case of reduced SOD2 activity, may enhance redox cycling allowing ferroptosis, inflammation and hearing dysfunctions [36, 84]. Moreover, aging and dysfunctions associated with ROS burden appear to have a great role in hearing deficit by accumulation of oxidative damage; therefore, antioxidant mechanisms are extremely useful in contrasting hearing deficit establishment, as supported by the effective antioxidant treatments in maintaining a healthy auditory system [84–86]. In detail, *SLC40A1* − 8CG is in complete linkage disequilibrium with *SLC40A1* -98GC, being both close to the iron regulatory element (IRE) target of the iron regulatory proteins (IRPs) in the promoter region of ferroportin with strong potential effects on gene expression via IRE/IRP interactions [75]. Similarly, *HAMP -*582AG in the promoter region of the hepcidin gene is located in a responsive element for upstream stimulatory factors (USF1/USF2) and − 582 A > G change let transcription factors not sufficiently bind the E-box leading to decrease transcription of the gene [87]. This is of particular interest considering its action as a negative feedback on the ferroportin internalization causing in turn iron dyshomeostasis/excess and unrestrained ROS production not properly counteracted by the presence of the loss of function gene variant *SOD2* 47C > T responsible for a decreased enzyme activity and a decreased neutralizing capacity of mitochondrial superoxide anion by 30–40% [80]. The *SOD2* 47T allele, responsible for the amino acid change A[GCT] > V[GTT], disrupts the α-helix structure of the enzyme essential for the enzyme translocation from the inner to the mitochondrial matrix.

Basically, investigations by single variable(s) approach just in part can explain the global complex mechanism responsible for SSNHL, and the hypothesized causative reasons altogether remind to unbalanced iron burden non optimally handled by an antioxidant pathway in which age, sex and global DNA methylation cooperate to the final clinical phenotype. In an explorative attempt, we investigated these variables by a cumulative statistic approach accounted by PCA to have a more realistic comprehensive picture. This tool is useful to reveal remote or subtle associations/among variables that may emerge in virtue of mutual additive or synergic interactions otherwise lost in single analyses due to non-statistically significant data involved in a definite observed clinical phenotype or in its severity progression. Interestingly, PCAs in the whole group yielded five principal components with different variables clustering, and PC1 was also significantly associated with the risk of progression to moderate/severe/profound HL as confirmed by further logistic regression analysis. In detail, PC1 mainly explains PTA, LINE-1 methylation and age, also including *HAMP* and *SLC40A1* variants. Moreover, these latter were mutually inversely related in the matrix components of PCA as well as methylation *versus* PTA or *versus* age supporting the hypothesis stemmed from single analyses referring that a misalignment of the factors involved in the iron burden management and oxidative stress may lead to SSNHL.

Finally, iron excess, suboptimal oxidative stress balance and anomalous DNA methylation may contribute to generate loss of function in sensitive cells, epithelium and organs, and then, epigenetic maintenance of the culprit organ and epigenetic age acceleration are reaching wide attention in order to recognize determinants associated with hearing loss of different origin as aging, occupational noise, drug treatments or idiopathic [53, 88–90]. Recognizing the etiopathogenesis of SSNHL at molecular level embraces great promise and will help to identify prognostic biomarkers and efficient therapeutic targets, as well as designing of novel epidrugs, inducing favorable epigenetic modulation to target and modulate epigenetic pathways or other mechanisms.

## Data Availability

All relevant data are within the manuscript.

## References

[1] Blazer DG (2020). Hearing loss: the silent risk for psychiatric disorders in late life. Clin Geriatr Med.

[2] Punch JL, Hitt R, Smith SW (2019). Hearing loss and quality of life. J Commun Disord.

[3] Mormer E, Cipkala-Gaffin J, Bubb K, Neal K (2017). Hearing and health outcomes: recognizing and addressing hearing loss in hospitalized older adults. Semin Hear.

[4] Korver AM, Smith RJ, Van Camp G, Schleiss MR, Bitner-Glindzicz MA, Lustig LR (2017). Congenital hearing loss. Nat Rev Dis Primers.

[5] Nirmalasari O, Mamo SK, Nieman CL, Simpson A, Zimmerman J, Nowrangi MA (2017). Age-related hearing loss in older adults with cognitive impairment. Int Psychogeriatr.

[6] Imtiaz F, Taibah K, Ramzan K, Bin-Khamis G, Kennedy S, Al-Mubarak B (2011). A comprehensive introduction to the genetic basis of non-syndromic hearing loss in the Saudi Arabian population. BMC Med Genet.

[7] Alexander TH, Harris JP (2013). Incidence of sudden sensorineural hearing loss. Otol Neurotol.

[8] Yoon CY, Kong TH, Lee J, Seo YJ, Ahn JJ (2023). Epidemiology of idiopathic sudden sensorineural hearing loss in the era of big data. Eur Arch Otorhinolaryngol.

[9] Koohiyan M (2020). Next generation sequencing and genetics of hereditary hearing loss in the iranian population: new insights from a systematic review. Int J Pediatr Otorhinolaryngol.

[10] Wells HRR, Newman TA, Williams FMK (2020). Genetics of age-related hearing loss. J Neurosci Res.

[11] Yan D, Xiang G, Chai X, Qing J, Shang H, Zou B (2017). Screening of deafness-causing DNA variants that are common in patients of European ancestry using a microarray-based approach. PLoS ONE.

[12] Cai XZ, Li Y, Xia L, Peng Y, He CF, Jiang L (2017). Exome sequencing identifies POU4F3 as the causative gene for a large Chinese family with non-syndromic hearing loss. J Hum Genet.

[13] Tang F, Choy E, Tu C, Hornicek F, Duan Z (2017). Therapeutic applications of histone deacetylase inhibitors in sarcoma. Cancer Treat Rev.

[14] Rehman AU, Friedman TB, Griffith AJ (2017). Unresolved questions regarding human hereditary deafness. Oral Dis.

[15] Xia W, Hu J, Liu F, Ma J, Sun S, Zhang J (2017). New role of LRP5, associated with nonsyndromic autosomal-recessive hereditary hearing loss. Hum Mutat.

[16] Paludetti G, Conti G, Din W, Dec E, Rolesi R, Picciotti PM (2012). Infant hearing loss: from diagnosis to therapy Official Report of XXI Conference of Italian Society of Pediatric Otorhinolaryngology. Acta Otorhinolaryngol Ital.

[17] http://hereditaryhearingloss.org/ [cited 2023 27/06/2023].

[18] Kremer H (2019). Hereditary hearing loss; about the known and the unknown. Hear Res.

[19] Brigande JV (2017). Hearing in the mouse of Usher. Nat Biotechnol.

[20] Mittal R, Aranke M, Debs LH, Nguyen D, Patel AP, Grati M (2017). Indispensable role of ion channels and transporters in the auditory system. J Cell Physiol.

[21] Yamada S, Kita J, Shinmura D, Nakamura Y, Sahara S, Misawa K (2022). Update on findings about sudden sensorineural hearing loss and insight into its pathogenesis. J Clin Med.

[22] Ricciardiello F, Pisani D, Viola P, Cristiano E, Scarpa A, Giannone A (2021). Sudden sensorineural hearing loss in mild COVID-19: case series and analysis of the literature. Audiol Res.

[23] Jeong J, Choi HS (2021). Sudden sensorineural hearing loss after COVID-19 vaccination. Int J Infect Dis.

[24] Singh AV, Vyas V, Montani E, Cartelli D, Parazzoli D, Oldani A (2012). Investigation of in vitro cytotoxicity of the redox state of ionic iron in neuroblastoma cells. J Neurosci Rural Pract.

[25] Singh AV, Subhashree L, Milani P, Gemmati D, Zamboni P (2010). Interplay of iron metallobiology, metalloproteinases, and FXIII, and role of their gene variants in venous leg ulcer. Int J Low Extrem Wounds.

[26] Galaris D, Barbouti A, Pantopoulos K (2019). Iron homeostasis and oxidative stress: an intimate relationship. Biochim Biophys Acta Mol Cell Res.

[27] Tisato V, Zuliani G, Vigliano M, Longo G, Franchini E, Secchiero P (2018). Gene-gene interactions among coding genes of iron-homeostasis proteins and APOE-alleles in cognitive impairment diseases. PLoS ONE.

[28] Gemmati D, Zeri G, Orioli E, De Gaetano FE, Salvi F, Bartolomei I (2012). Polymorphisms in the genes coding for iron binding and transporting proteins are associated with disability, severity, and early progression in multiple sclerosis. BMC Med Genet.

[29] Gemmati D, Tognazzo S, Catozzi L, Federici F, De Palma M, Gianesini S (2006). Influence of gene polymorphisms in ulcer healing process after superficial venous surgery. J Vasc Surg.

[30] Tognazzo S, Gemmati D, Palazzo A, Catozzi L, Carandina S, Legnaro A (2006). Prognostic role of factor XIII gene variants in nonhealing venous leg ulcers. J Vasc Surg.

[31] Zamboni P, Gemmati D (2007). Clinical implications of gene polymorphisms in venous leg ulcer: a model in tissue injury and reparative process. Thromb Haemost.

[32] Castiglione A, Ciorba A, Aimoni C, Orioli E, Zeri G, Vigliano M (2015). Sudden sensorineural hearing loss and polymorphisms in iron homeostasis genes: new insights from a case-control study. Biomed Res Int.

[33] Chien CY, Huang TY, Tai SY, Chang NC, Wang HM, Wang LF (2017). Glutathione peroxidase 3 gene polymorphisms and the risk of sudden sensorineural hearing loss. Kaohsiung J Med Sci.

[34] Kitoh R, Nishio SY, Ogawa K, Okamoto M, Kitamura K, Gyo K (2016). SOD1 gene polymorphisms in sudden sensorineural hearing loss. Acta Otolaryngol.

[35] Teranishi M, Uchida Y, Nishio N, Kato K, Otake H, Yoshida T (2012). Polymorphisms in genes involved in oxidative stress response in patients with sudden sensorineural hearing loss and Meniere's disease in a Japanese population. DNA Cell Biol.

[36] Sun Y, Zou S, He Z, Chen X (2022). The role of autophagy and ferroptosis in sensorineural hearing loss. Front Neurosci.

[37] Gemmati D, Longo G, Gallo I, Silva JA, Secchiero P, Zauli G (2022). Host genetics impact on SARS-CoV-2 vaccine-induced immunoglobulin levels and dynamics: the role of TP53, ABO, APOE, ACE2, HLA-A, and CRP genes. Front Genet.

[38] Boison D, Rho JM (2020). Epigenetics and epilepsy prevention: The therapeutic potential of adenosine and metabolic therapies. Neuropharmacology.

[39] Tisato V, Zauli G, Rimondi E, Gianesini S, Brunelli L, Menegatti E (2013). Inhibitory effect of natural anti-inflammatory compounds on cytokines released by chronic venous disease patient-derived endothelial cells. Mediators Inflamm.

[40] Obri A, Serra D, Herrero L, Mera P (2020). The role of epigenetics in the development of obesity. Biochem Pharmacol.

[41] Shamsi MB, Firoz AS, Imam SN, Alzaman N, Samman MA (2017). Epigenetics of human diseases and scope in future therapeutics. J Taibah Univ Med Sci.

[42] Gemmati D, Tisato V, Legato MJ, Feldberg D, Glezerman M (2023). Chapter 15—genetics and epigenetics of the one-carbon metabolism pathway in autism spectrum disorder: Role of a sex-specific brain epigenome. Sex, Gender, and Epigenetics.

[43] Gemmati D, Tisato V, Legato MJ (2023). Chapter 24—genomic and epigenomic signature at the branch-point among genome, phenome, and sexome in health and disease: a multiomics approach. Principles of gender-specific medicine.

[44] Tisato V, Silva JA, Longo G, Gallo I, Singh AV, Milani D (2021). Genetics and epigenetics of one-carbon metabolism pathway in autism spectrum disorder: a sex-specific brain epigenome?. Genes (Basel)..

[45] Zhou Q, Xiong Y, Qu B, Bao A, Zhang Y (2021). dna methylation and recurrent pregnancy loss: a mysterious compass?. Front Immunol.

[46] Gemmati D, Tognazzo S, Serino ML, Fogato L, Carandina S, De Palma M (2004). Factor XIII V34L polymorphism modulates the risk of chronic venous leg ulcer progression and extension. Wound Repair Regen.

[47] Zhao Q, Ge Z, Fu S, Wan S, Shi J, Wu Y (2022). DNA methylation plays an important role in iron-overloaded Tibetans. Genes Genet Syst.

[48] Yamamoto M, Tanaka H, Toki Y, Hatayama M, Ito S, Addo L (2016). Iron-induced epigenetic abnormalities of mouse bone marrow through aberrant activation of aconitase and isocitrate dehydrogenase. Int J Hematol.

[49] Heinsberg LW, Weeks DE, Alexander SA, Minster RL, Sherwood PR, Poloyac SM (2021). Iron homeostasis pathway DNA methylation trajectories reveal a role for STEAP3 metalloreductase in patient outcomes after aneurysmal subarachnoid hemorrhage. Epigenetics Commun..

[50] Sharp PA, Clarkson R, Hussain A, Weeks RJ, Morison IM (2018). DNA methylation of hepatic iron sensing genes and the regulation of hepcidin expression. PLoS ONE.

[51] Stankiewicz AM, Swiergiel AH, Lisowski P (2013). Epigenetics of stress adaptations in the brain. Brain Res Bull.

[52] Guo L, Li PH, Li H, Colicino E, Colicino S, Wen Y (2017). Effects of environmental noise exposure on DNA methylation in the brain and metabolic health. Environ Res.

[53] Guo L, Wang W, Song W, Cao H, Tian H, Wang Z (2023). Genome-wide DNA methylation analysis of middle-aged and elderly monozygotic twins with age-related hearing loss in Qingdao. China Gene.

[54] Marques-Rocha JL, Milagro FI, Mansego ML, Mourao DM, Martinez JA, Bressan J (2016). LINE-1 methylation is positively associated with healthier lifestyle but inversely related to body fat mass in healthy young individuals. Epigenetics.

[55] Tajuddin SM, Amaral AF, Fernandez AF, Rodriguez-Rodero S, Rodriguez RM, Moore LE (2013). Genetic and non-genetic predictors of LINE-1 methylation in leukocyte DNA. Environ Health Perspect.

[56] Seker Yildiz K, Durmus K, Donmez G, Arslan S, Altuntas EE (2017). Studying the association between sudden hearing loss and DNA N-methyltransferase 1 (DNMT1) genetic polymorphism. J Int Adv Otol.

[57] Ma PW, Wang WL, Chen JW, Yuan H, Lu PH, Gao W (2022). Treatment with the ferroptosis inhibitor ferrostatin-1 attenuates noise-induced hearing loss by suppressing ferroptosis and apoptosis. Oxid Med Cell Longev.

[58] Gemmati D, Castiglione A, Vigliano M, Ciorba A, Aimoni C. Sudden sensorineural hearing loss and polymorphisms in iron homeostasis genes. In: Handbook of Hearing Disorders Research. 2015. p. 77–84. 10.1155/2015/834736 PMC4348611 25789325

[59] Ciorba A, Bianchini C, Crema L, Ceruti S, Ermili F, Aimoni C (2019). White matter lesions and sudden sensorineural hearing loss. J Clin Neurosci.

[60] Corazzi V, Ciorba A, Bianchini C, Pelucchi S, Skarzynski PH, Hatzopoulos S (2021). Genetic polymorphisms in sudden sensorineural hearing loss: an update. Ear Nose Throat J.

[61] Castiglione A, Casa M, Gallo S, Sorrentino F, Dhima S, Cilia D (2019). Correspondence between cognitive and audiological evaluations among the elderly: a preliminary report of an audiological screening model of subjects at risk of cognitive decline with slight to moderate hearing loss. Front Neurosci.

[62] Gemmati D, Longo G, Franchini E, Araujo Silva J, Gallo I, Lunghi B (2021). Cis-segregation of c.1171C>T stop codon (p.R391*) in SERPINC1 gene and c.1691G>A transition (p.R506Q) in F5 gene and selected GWAS multilocus approach in inherited thrombophilia. Genes (Basel)..

[63] Haberkamp TJ, Tanyeri HM (1999). Management of idiopathic sudden sensorineural hearing loss. Am J Otol.

[64] Marchetti G, Gemmati D, Patracchini P, Pinotti M, Bernardi F (1991). PCR detection of a repeat polymorphism within the F7 gene. Nucleic Acids Res.

[65] Fallah F, Colagar AH, Saleh HA, Ranjbar M (2023). Variation of the genes encoding antioxidant enzymes SOD2 (rs4880), GPX1 (rs1050450), and CAT (rs1001179) and susceptibility to male infertility: a genetic association study and in silico analysis. Environ Sci Pollut Res Int.

[66] Mittal R, Bencie N, Liu G, Eshraghi N, Nisenbaum E, Blanton SH (2020). Recent advancements in understanding the role of epigenetics in the auditory system. Gene.

[67] Dinh CT, Nisenbaum E, Chyou D, Misztal C, Yan D, Mittal R (2020). Genomics, epigenetics, and hearing loss in neurofibromatosis type 2. Otol Neurotol.

[68] Martini A, Sorrentino F, Sorrentino U, Cassina M (2021). Genetics & epigenetics of hereditary deafness: an historical overview. Audiol Res.

[69] Cui Y, Liang W, Li M, Zhao Z, Jiang X, Zhao B (2022). Better late than never: initial experience of intra-arterial pulsed-urokinase-injection as a salvage therapy for refractory sudden sensorineural hearing loss. Interv Neuroradiol.

[70] Passamonti SM, Di Berardino F, Bucciarelli P, Berto V, Artoni A, Gianniello F (2015). Risk factors for idiopathic sudden sensorineural hearing loss and their association with clinical outcome. Thromb Res.

[71] Berrettini S, Seccia V, Fortunato S, Forli F, Bruschini L, Piaggi P (2013). Analysis of the 3-dimensional fluid-attenuated inversion-recovery (3D-FLAIR) sequence in idiopathic sudden sensorineural hearing loss. JAMA Otolaryngol Head Neck Surg.

[72] Shi X (2016). Pathophysiology of the cochlear intrastrial fluid-blood barrier (review). Hear Res.

[73] da Silva VK, de Freitas BS, Dornelles VC, Kist LW, Bogo MR, Silva MC (2018). Novel insights into mitochondrial molecular targets of iron-induced neurodegeneration: reversal by cannabidiol. Brain Res Bull.

[74] Mei H, Zhao L, Li W, Zheng Z, Tang D, Lu X (2020). Inhibition of ferroptosis protects House Ear Institute-Organ of Corti 1 cells and cochlear hair cells from cisplatin-induced ototoxicity. J Cell Mol Med.

[75] Lee PL, Gelbart T, West C, Halloran C, Felitti V, Beutler E (2001). A study of genes that may modulate the expression of hereditary hemochromatosis: transferrin receptor-1, ferroportin, ceruloplasmin, ferritin light and heavy chains, iron regulatory proteins (IRP)-1 and -2, and hepcidin. Blood Cells Mol Dis.

[76] Lim D, Kim KS, Jeong JH, Marques O, Kim HJ, Song M (2018). The hepcidin-ferroportin axis controls the iron content of Salmonella-containing vacuoles in macrophages. Nat Commun.

[77] El-Gharbawi N, Shaheen I, Hamdy M, Elgawhary S, Samir M, Hanna BM (2023). Genetic Variations of ferroportin-1(FPN1-8CG), TMPRSS6 (rs855791) and Hemojuvelin (I222N and G320V) Among a Cohort of Egyptian beta-Thalassemia Major Patients. Indian J Hematol Blood Transfus.

[78] Zarghamian P, Azarkeivan A, Arabkhazaeli A, Mardani A, Shahabi M (2020). Hepcidin gene polymorphisms and iron overload in beta-thalassemia major patients refractory to iron chelating therapy. BMC Med Genet.

[79] Valenti L, Conte D, Piperno A, Dongiovanni P, Fracanzani AL, Fraquelli M (2004). The mitochondrial superoxide dismutase A16V polymorphism in the cardiomyopathy associated with hereditary haemochromatosis. J Med Genet.

[80] Sutton A, Khoury H, Prip-Buus C, Cepanec C, Pessayre D, Degoul F (2003). The Ala16Val genetic dimorphism modulates the import of human manganese superoxide dismutase into rat liver mitochondria. Pharmacogenetics.

[81] Singh AV, Chandrasekar V, Paudel N, Laux P, Luch A, Gemmati D (2023). Integrative toxicogenomics: advancing precision medicine and toxicology through artificial intelligence and OMICs technology. Biomed Pharmacother.

[82] Cervellati C, Valacchi G, Tisato V, Zuliani G, Marsillach J (2019). Evaluating the link between Paraoxonase-1 levels and Alzheimer's disease development. Minerva Med.

[83] Slusarczyk P, Mleczko-Sanecka K (2021). The multiple facets of iron recycling. Genes (Basel)..

[84] Jamesdaniel S, Ramkumar V, Rybak LP (2018). Oxidative stress and hearing loss. Inflammatory mechanisms in mediating hearing loss.

[85] Bermudez-Munoz JM, Celaya AM, Hijazo-Pechero S, Wang J, Serrano M, Varela-Nieto I (2020). G6PD overexpression protects from oxidative stress and age-related hearing loss. Aging Cell.

[86] Varela-Nieto I, Murillo-Cuesta S, Rodriguez-de la Rosa L, Oset-Gasque MJ, Marco-Contelles J (2021). Use of radical oxygen species scavenger nitrones to treat oxidative stress-mediated hearing loss: state of the art and challenges. Front Cell Neurosci.

[87] Parajes S, Gonzalez-Quintela A, Campos J, Quinteiro C, Dominguez F, Loidi L (2010). Genetic study of the hepcidin gene (HAMP) promoter and functional analysis of the c.-582A > G variant. BMC Genet.

[88] Kuo PL, Moore AZ, Lin FR, Ferrucci L (2021). Epigenetic age acceleration and hearing: observations from the Baltimore longitudinal study of aging. Front Aging Neurosci.

[89] Zhang Y, Huang S, Dai X, Xia ZF, Xiao H, He XL (2021). SOD2 alleviates hearing loss induced by noise and kanamycin in mitochondrial DNA4834-deficient rats by regulating PI3K/MAPK signaling. Curr Med Sci.

[90] Moos WH, Faller DV, Glavas IP, Harpp DN, Irwin MH, Kanara I (2018). A new approach to treating neurodegenerative otologic disorders. Bioresour Open Access.

[91] Tisato V, Muggeo P, Lupiano T, Longo G, Serino ML, Grassi M, Arcamone E, Secchiero P, Zauli G, Santoro N (2019). Maternal haplotypes in DHFR promoter and MTHFR gene in tuning childhood acute lymphoblastic leukemia onset-latency: Genetic/Epigenetic Mother/Child Dyad Study (GEMCDS). Genes..

